# Manganese as an Emmenagogue*Read before the Chicago Medical Society, January 5, 1885.

**Published:** 1885-02

**Authors:** Franklin H. Martin

**Affiliations:** 22d Street and Wabash Avenue; Chicago


### ARTICLE III.

MANGANESE AS AN EMMENAGOGUE.* *By* FRANKLIN H. MARTIN, M. D., *Chicago.*

Ringer and Murrell, of London, in a little article (*Lancet*, Jan. 6, 1883) called the attention of the profession to gratify-

*Read before the Chicago Medical Society, January 5, 1885.

ing results obtained in experiments performed by them in the treatment of amenorrhœa by the salts of manganese. Since then I have taken advantage of every opportunity afforded me in dispensary and private practice to satisfy myself as to the action and efficacy of the new remedy in this direction. I published the results of my first investigations in the *New York Record*, Sept. 29th, 1883.*

Since the publication of that article I have continued my investigations with considerable zeal and now wish to give additional testimony confirming the efficacy of the remedy as a menstrual stimulant.

I have found that manganese will not only relieve certain forms of *amenorrhœa* but will also relieve forms of *menorrhagia* and *metrorrhagia*. As these several conditions, AMENORRHŒA, menorrhagia and metrorrhagia, are dependent upon so many different causes, it is very necessary for us to discriminately point out the exact conditions in which manganese is indicated. Ringer and Murrell in recommending manganese for amenorrhœa neglected to point out with explicitness the peculiar forms of amenorrhœa in which they found their remedy to exert its greatest influence.

"From my observation I have been led to consider manganese in any form a direct stimulant to the uterus and its appendages. It may exert this influence by acting as direct vasomotor nerve stimulant to the vascular system of the parts, and in consequence of the improved circulation directly increase the tone and nutrition of the organ, as it may exert its whole force through stimulation of the sexual nerve ganglia, or even possibly the sexual nerve centers, thereby bringing the organs to their normal state of action." At any rate its action is

---

*When not otherwise indicated, quotations in this article are from my article on manganese that was published in the *New York Record*, Sept. 29th, 1883—M.

prompt, and direct in bringing the uterus and appendages to a normal state of menstrual tonicity, when the lack of tone is dependent upon some previous depression of innervation.

It will be seen by the following typical cases, that even when the cause of the depressed innervation is still acting, this remedy will exert its stimulating power over the menstrual mechanism. A young woman, eighteen years of age, in consequence of phthisis had not menstruated in four months. Experimentally I gave her manganese. Menstruation occurred within a week. "Another young woman, twenty-four years of age, with an aggravating digestive trouble of some years' standing, had become very irregular—flowing profusely for a week or two, then scantily for an equally irregular time, again followed, perhaps without warning, by a profuse flow or as likely a complete cessation. This state of affairs had been going on for more than a year. There was no dysmenorrhœa. She was very weak and anæmic from the effects of indigestion and loss of blood. This patient was given two grains of the permanganate of potash dissolved in one-half glass of hot water every night on retiring. It was kindly received in this way by the irritable digestive organs. In a very short time there was a decided improvement in the menstrual trouble, and the patient has since menstruated three times normally."

"In young girls who are irregular in the early months of menstrual life, where it is simply caused by the natural weakness of the partially developed organs of generation; or where, from an overworked nervous system, the organs are robbed of their natural nerve force, this remedy seems to possess the stimulating properties requisite to bring them into healthy action. A remarkable case of this kind was that of a young girl who had menstruated once. Eight months had passed and the menstrual flow had failed to appear again. The mother of the girl being alarmed sought advice. The permanganate was given in two grain doses twice a day. Within a week the girl menstruated the second time in her life. In two other cases of 'missing' in young girls, without any apparent cause, or any other symptoms, the remedy given in the same doses a few days before the next regular period was expected, stimulated the organs to a normal flow. The action of the manganese was so prompt in these cases that I am convinced it was no coincidence." Since the above report was made I have been able to add many similar cases to my list, and the results have been invariably as gratifying.

" It is well known that from exposure to cold the weakest organs of the body are the ones most liable to suffer. A woman who, when exposed to cold, immediately suffers suppression, cessation, or excess of the menstrual flow, will invariably be found to possess susceptible and weak menstrual organs. In cases of this kind, viz.: suppression, cessation or excess of menstrual flow, caused by catching cold, with no other apparent cause, the most gratifying and prompt results are obtained from manganese. The above variety of cases are of so frequent occurrence that in them I have had numerous opportunities to test the new remedy, and I have yet to see it fail, in either amenorrhœa or menorrhagia, when due to irritation of cold alone. In several cases where the flow was a week or ten days overdue from catching cold, the permanganate was given in large doses, and its almost magical effect demonstrated by the flow appearing within twelve hours."

My principle object in making *this report* is to emphasize the fact that manganese is a general stimulant of the menstrual organ, and in consequence of its stimulating power is an efficient remedy in certain forms of *menorrhagia* and *metrorrhagia.* In my report one year ago, I mentioned this point, but at that time my opportunities for testing the drug in that direction had been somewhat limited. I said at that time:

"Although I have had greater opportunities for testing the value of manganese in *amenorrhœa* than in *menorrhagia* or *metrorrhagia*, I have received unmistakable evidence of its power in the latter forms of menstrual trouble."

"Menorrhagia and amenorrhœa in their outer manifestations are exactly opposite in nature, but they are very often dependent upon the same causes. When the cause is anæmia, or any depressing constitutional disease, producing a perversion of the functional activity of the menstrual organs, and their *perverted* action consists of an *irregular* or *excessive* flow, this condition will as readily succumb to the stimulating effects of manganese as when the opposite condition exists. The following cases are of interest: a woman, aged twenty-six, sought advice for excessive and irregular flowing. She had been married two years, and had one child, twelve months old. The child was large and strong, the mother physically slight. The mother nursed the child, and for ten months stood the strain very well, when she commenced to fail, suddenly grew weak and anæmic and began to flow excessively. This continued with but a few short irregular remissions until I saw her at the dispensary. She was given two-grain-doses of the permanganate of potash four times a day. At the same time all other treatment was withheld. In three days the patient returned, saying that the flow had stopped the next day after receiving her medicine. I then discontinued the manganese, prescribed iron and nourishing food, and she continued to improve. By digital examination nothing abnormal was revealed in the above case. Another case was that of a large, stout woman, thirty-five years of age, who came to the dispensary suffering from menorrhagia. Her menstrual periods were regular as to *time*, but the quantity of blood was alarmingly excessive, and would last for two weeks. She was married, had three children, the youngest three years of age. This abnormal condition of menstruation had been coming on by degrees for a year. The uterus was a little enlarged, and soft to the touch; otherwise, by physical examination, nothing abnormal. Four days before the expected flow, she commenced taking the permanganate in two-grain doses, three times a day. Menstruation came on at the expected time, and after a normally free flow for four days, passed off naturally. Before the next period the same treatment was repeated, with the same marvellous result."

The above histories were written a year ago. Since then I have seen a great many cases of menorrhagia and metrorrhagia succumb to the effects of manganese. I have complete histories of a number of these cases which might be of interest, but will be content to mention a few typical cases taken from the case book of Dr. S. F. Bradley, of this city, a gentleman who has taken considerable pains to test the value of manganese in menstrual troubles.

I. "Mrs. D——, age thirty-three. Treated during winter and spring of 1883 for chronic endo-cervicitis and endo-metritis; and enjoyed good health until August 10th, when she was taken with menorrhagia, which lasted six days before medical advice was sought. Gave in succession ergot, gallic acid, hot injections, injected tincture of iodine into uterine cavity, and finally plugged cervical canal with sponge tent which remained twenty hours. Hæmorrhage still continued. Then ordered capsules, each containing two-and-one-half grains of permanganate of potash, one to be taken every three hours. Flow ceased shortly after taking the second capsule.

II. "Mrs. F——, aged thirty. Made first visit January 19th,

1884. Three weeks before had a miscarriage at three months, and was attended by a midwife. January 19th, began flowing excessively, and suffered much pain in uterine region. Detected by digital examination a piece of placental tissue about size of hen's egg in uterine cavity. Removed with fingers, and flow ceased. From this time until February 24th, she improved rapidly from her anemic condition, but at the above date menorrhagia began and was excessive. Ergot (℥j fl. extract every two hours) was given during the night with no cessation of flow. The following day ordered two-grain capsules of permanganate of potash. Hæmorrhage ceased in two hours after taking first capsule.

Case III. "Mrs. McF——, age thirty. Two children, youngest one and a half year old. Began flowing in the middle of Sept., 1883. She sought no medical advice until Oct. 15th. The hæmorrhage had rendered her exceedingly weak and anemic. Gave ergot and elixir ferri quin. et strych. She continued this prescription for ten days, with no benefit. Ergot and gallic acid were then used with no amelioration. Careful examination revealed no enlargement of uterus—no tenderness—very slight laceration of cervix. On the 28th of Oct. ordered two-grain capsules of permanganate of potash, one every four hours. One hour after first capsule had been taken the flow ceased suddenly. The capsules were continued until three or four had been taken. Saw her a few weeks ago and her health had been excellent.

Case IV. "Mrs. B——, age thirty-five. Five children, youngest two years. Laceration of cervix (bilateral). Uterus four inches in depth. Chronic endo-cervicitis and endometritis—slight prolapsus. Treated her for uterine trouble a couple of months previously, and had advised operation. During the middle of October was taken unwell, flow never so profuse. Had continued several days before seeking medical advice. Ergot, mineral and vegetable astringents had no effect. Gave the permanganate in two-grain doses. Shortly after first capsule was adminstered the flow began to diminish, and ceased entirely after the third had been given."

As has been demonstrated in the foregoing cases, the action of the drug is very prompt where indicated, and if discriminately given I am sure similar gratifying results will be obtained by others.

Dr. T. Gaillard Thomas, in an address on obstetrics and gynæcology, delivered at the first annual meeting of the New York State Medical Association, and published in the *Medical News*, of Philadelphia (Nov. 22), said of this remedy: "Permanganate of potash . . . . . . . . . as an excitant of the menstrual flow is, I think, the best emmenagogue which has yet been discovered." Dr. Roberts Bartholow, in a recent article on permanganate of potash, said: "The powers possessed by permanganate of potassium as a general stimulant are well exhibited in the active emmenagogue property which it has been shown to possess by Drs. Ringer and Murrell. In cases of amenorrhœa, due to deficient activity, it seems to promote the function in a remarkable degree. The same power which can so stimulate the sexual functions must, when exerted in other directions, prove equally effective."

"Although manganese, like the allied metals, nickel, zinc, iron and silver, has a direct influence on the blood as a tonic in anæmia, chlorosis, etc., it cannot be possible, in my opinion, that its peculiar influence on the catemenia can alone depend upon that virtue. To influence the organs of menstruation by acting as a general tonic, would necessarily be a slow process, and the effect would be very gradual. It would undoubtedly, however, as a general tonic have a predilection for these organs. This was noticed and commented upon by W. H. Broadbent, of London, after experiments performed by him and recorded in the " Proceedings " of the Clinical Society, of London, for 1868-69, Vol. II., p. 122. 'Manganese,' he says, 'seemed to have a special influence in promoting the returu of the catamenia and nickel a special property of checking leucorrhœa.' But one can readily see by the character of the cases reported this evening, that manganese must have a more direct mode of influencing the menstrual organs than by the necessarily slow one of a general tonic."

In prescribing manganese it is well to bear in mind a few points of importance. Permanganate of potash (the original preparation used) has a disagreeable, distressing effect on the stomach when taken undiluted, which may be obviated by administering on a full stomach—immediately after eating—or dissolved in considerable water. In administering the permanganate in pill form it must be remembered that excipients ordinarily used by dispensers will produce with the drug spontaneous combustion. Dry gelatine capsules I have found to be the most convenient form in which to administer this preparation.

Dr. Roberts Bartholow says of permanganate of potash, in regard to mode of prescribing : " As this salt is so readily decomposed, yielding up its oxygen to any organic matter present, it is obviously necessary to be very careful in preparing and administering it. It should be given dissolved in pure water, or in compressed tablets. These compressed tablets, made by Wyeth & Brother, of Philadelphia, contain no excip ient, and are, therefore, entirely free from objection,"

22d Street and Wabash Avenue.

